# An Exploration of the Relations between External Representations and Working Memory

**DOI:** 10.1371/journal.pone.0006513

**Published:** 2009-08-04

**Authors:** Jiajie Zhang, Hongbin Wang

**Affiliations:** School of Health Information Sciences, University of Texas Health Science Center at Houston, Houston, Texas, United States of America; University of Sydney, Australia

## Abstract

It is commonly hypothesized that external representations serve as memory aids and improve task performance by means of expanding the limited capacity of working memory. However, very few studies have directly examined this memory aid hypothesis. By systematically manipulating how information is available externally versus internally in a sequential number comparison task, three experiments were designed to investigate the relation between external representations and working memory. The experimental results show that when the task requires information from both external representations and working memory, it is the interaction of information from the two sources that determines task performance. In particular, when information from the two sources does not match well, external representations hinder instead of enhance task performance. The study highlights the important role the coordination among different representations plays in distributed cognition. The general relations between external representations and working memory are discussed.

## Introduction

Despite the limited capacity of working memory, people can perform complex cognitive tasks fairly well when they interact with external representations – those representations that exist outside the human mind. For example, multiplying 3735 by 9278 using paper and pencil is a much easier job than doing it in the head. One common explanation is the *memory aid hypothesis*: external representations help problem solving by augmenting the limited-capacity working memory with a much larger external memory storage [e.g.], [ 1], [2]–[4]. As a result, some information can reside in the external memory storage and be picked up and represented in appropriate forms by the cognitive system only when necessary.

While intuitively appealing, this hypothesis has been challenged. In the seminal paper by Larkin and Simon [5] it has been suggested that representations and processes are both important in cognitive performance and that one cannot talk about representations without talking about processes that operate on representations. In addition, a large body of research in the field of distributed cognition suggests that the role of external representations goes beyond the memory aid. For example, it has been found that not only the amount but also the formats of external representations affect cognitive performance. The latter is called the representational effect [6]–[8], which is especially puzzling in the sense that it implies that external representations may be more deeply involved in cognitive problem solving than simply providing a memory aid. On the other hand, Zhang and colleagues have suggested that external representations need not be re-represented as internal representations in order to be used in problem solving: they can directly activate perceptual processes and directly provide perceptual information, which, in conjunction with internal representations and cognitive processes, determine the behavior [e.g.], [ 6]–[8], [9], [10], [11]. According to this view, in cognitive tasks that involve external representations, the behavior is the integrative processing of the information perceived from external representations and that retrieved from internal representations through the interplay of perceptual and cognitive processes.

However, how the interplay works raises an issue. One essential aspect of the issue has to do with how people coordinate the use of external representations and internal representations, an essential aspect of cognitive executive function. Apparently, if the coordination is poor, even with a large amount of external representations as the memory aid to working memory, cognitive performance may not be improved.

In the present study we conducted three experiments to explore the issue. We adopted a sequential number comparison task. By systematically manipulating how information is available externally versus internally, our results show that external representations can hinder as well as enhance task performance, illustrating the important role the coordination among different representations plays in distributed cognitive problem solving.

## Methods and Results

The experimental task of the present study is based on the list-processing task originally developed by Weber, Burt, and Noll [12] and later modified by Dark [13] and Carlson, Wenger, and Sullivan [14], [15]. In this task, participants are asked to mix two lists (e.g., B-F-C and M-L-G) to generate a new list by alternating items from each list (e.g., B-M-F-L-C-G). One interesting manipulation is where the two lists are when the mixing is being conducted: they can be available internally (i.e., retrieved from memory based on earlier studies), or externally (i.e., visible on a screen). The first study using this task by Weber, Burt, and Noll [12] showed that tasks with one list available internally and the other one available externally were more time-consuming and error-prone than those with the lists available both internally or both externally. The authors attributed this result to the extra cost of switching attention across internal memory and external presentations. However, a later study by Dark [13] indicated that the difficulty of the task was more from memory retrieval rather than attention switching. The study by Carlson, Wenger, and Sullivan [14], [15] showed that the critical factor in the list-processing task was the coordination of activities, which depended on the means available for storing and generating sequences.

**Figure 1:**
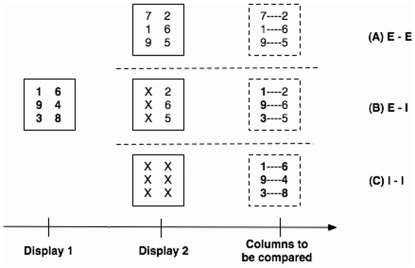
Examples of experimental trials. Participants are required to memorize the digits in Display 1 and, when seeing Display 2, respond by comparing the magnitude of digits row-by-row. Depending on the conditions, the to-be-compared columns may come from Display 2 (A), Display 1 and 2 (B), and Display 1 (C).

In the present study we slightly modify the original list-processing task in order to examine the interplay of external and internal representations in distributed problem solving. In this task, we ask participants to compare the magnitude of the corresponding digits in two digit columns (see Figure 1). Each trial consists of two displays. In Display 1 we present the two digit columns with two or three digits in each column. The participants are simply instructed to memorize these digits. After Display 1 disappears, Display 2 is presented at the same screen location and stays visible until all responses are made. Display 2 consists of two columns of digits and/or X's., resulting in three possible conditions: 1) Display 2 has two columns of digits (Figure 1A). Therefore the task is to simply compare the two columns on Display 2 (i.e., ignoring Display 1. We call this condition “E-E”, indicating that both columns of to-be-compared digits are externally available; 2) Display 2 has one column of digits and one column of X's (Figure 1B). The task is to compare the column of digits on Display 2 with the other column of digits on Display 1. We call this condition “E-I”, indicating that one column of to-be-compared digits is available externally and another column is available internally; and 3) Display 2 has two columns of X's (Figure 1C). The task is to compare the two memorized columns on Display 1 (i.e., ignoring Display 2). We call this condition “I-I”, indicating that both columns of to-be-compared digits have to be retrieved internally from memory. In all conditions, participants are required to compare the corresponding digits row-by-row from the top to the bottom as soon as Display 2 appears, by pressing the left key if the left digit is larger and the right key if the right digit is larger.

To successfully perform the sequential comparison task, participants need to know the to-be-compared digits and their positions, which can be available either externally (direct perception) or internally (memory retrieval). By manipulating how information is distributed across external and internal representations and the encoding instructions for Display 1, we designed three experiments. All three experiments used the same three representation types (*E-E, E-I, I-I*) shown in Figure 1 but differed in the instructions given for memorizing Display 1. In Experiment 1, participants were instructed to memorize the digits on Display 1 *column by column*, that is, from the top to the bottom on the left column, then from the top to the bottom on the right column. In Experiment 2, participants were instructed to memorize the digits on Display 1 *column by column in a reversed order*, that is, from the bottom to the top on the left column, then from the bottom to the top on the right column. In Experiment 3, participants were instructed to memorize the digits on Display 1 *row by row*, that is, memorize the top row first (from left to right), then middle row (if there are three digits in each column) and bottom row. In all three experiments, however, the response patterns that participants needed to make were always the same, that is, always from the top to the bottom pair. In each trial, we measured the accuracy and reaction time of the participant's key responses.

One additional factor we manipulated in our experiments is the number of digits per column (column length). By making column length to be either 2 or 3, we controlled the number of items to be maintained in working memory after Display 1 to be 4 or 6. Considering the limited capacity of working memory, this manipulation allows us to examine whether the load of working memory has an effect on its interaction with external representations.

The purposes of these experiments are two-fold. First, we would like to explore whether a task always becomes easier when more information is in external representations and less information is in internal representations. If it is so, one would expect the *I-I* condition is harder than the *E-I* condition, which is harder than the *E-E* condition, regardless of the encoding instructions given for Display 1. Second, we would like to explore how different representations are coordinated and how digits in different representations are matched and compared. Presumably, different encoding instructions for Display 1 lead to different internal representations of those digits. However, the digits (or X's) on Display 2 are always visible and fixed as external representations. Comparing and contrasting different conditions with different internal representations and fixed external representations allows us to systematically examine the interplay of different representations in performing the task.

### Experiment 1

In this experiment, participants were instructed to memorize the digits on Display 1 *column by column*, that is, from the top to the bottom on the left column, then from the top to the bottom on the right column. This encoding strategy results in a memorized list ordered in such a way that it benefits *E-I* comparisons. For example, if the left column on Display 1 is to be compared with the right column on Display 2, all participants need to do is to retrieve the first 2 or 3 digits in the memorized list and perform the comparison in order. In contrast, if the comparison is among memorized digits (i.e., *I-I*), this encoding strategy hurts the performance in that participants need to jump back and forth in the memorized list.

#### Participants

24 undergraduate students in introductory psychology courses at The Ohio State University participated in the experiment for course credit. The study was approved by the institutional review board of The Ohio State University and written informed consent was obtained from each participant.

#### Stimuli and Procedure

Participants were seated about 50 cm from the computer monitors. The digits and X's were in 24 point New York font, with a horizontal inter-digit distance of 2.5 cm and a vertical inter-digit distance of 1.0 cm. Each trial consisted of two displays, preceded by a ‘+’ sign for 500 ms for fixation. Display 1 had two columns of digits with two or three digits in each column, being presented for two seconds for cases with two digits per column and three seconds for cases with three digits per column (i.e., 500 ms per digit). The participants were instructed to memorize these two columns of digits *column by column*, that is, memorize the left column first from the top digit to the bottom digit, then memorize the right column from the top digit to the bottom digit. One second after Display 1 disappeared, Display 2 was presented at the same location and stayed visible on the screen until all responses were made or until it was over ten seconds.

Participants were instructed to compare the magnitudes of the two target columns from the top to the bottom pair (row) as soon as Display 2 appeared, by pressing the left key if the left digit was larger and the right key if the right digit was larger. Both speed and accuracy were emphasized. The total reaction times (RTs), participants' decisions, and errors were recorded. The total RT for each trial was also decomposed into individual RTs: the RT for the first comparison was the latency from the onset of Display 2 until the first response; the RT for the second comparison was from the first to the second response; and the RT for the third comparison (for 3-digit columns only) was from the second to the third response.

#### Design

A mixed design was used. The between-subject factor was column length: 2 and 3 digits per column. The within-subject factor was representation: *E-E*, *I-I*, and *E-I*. Both length 2 and length 3 conditions had forty-eight trials, with sixteen in each representation type. The forty-eight trials were completely randomized for each participant. Twelve participants received forty-eight length 2 trials, and twelve other participants received forty-eight length 3 trials. Every pair of digits to be compared had the same numerical distance of five (e.g., 2 vs. 7) to reduce the variance caused by the distance effect of number comparisons [16], [17]. The pairs of digits to be compared were randomized, with the constraint that the pairs in any given trial were all different.

#### Results

Trials with errors were excluded from the analyses of RTs. An error occurred if one or more responses of a trial were incorrect. For each participant, the RTs for the sixteen trials for each representation type, after the removal of outliers that deviated from the mean by two standard deviations, were pooled for statistical analyses.

The error rates for *E-E*, *E-I*, and *I-I* were 0.52%, 5.2%, and 5.7% for length 2, and 3.1%, 8.9%, and 4.2% for length 3. A two-way ANOVA for the three representations and two column lengths showed a non-significant interaction, a non-significant length effect, but a significant representation effect (F(2, 44) = 6.08, p<0.01). Simple comparisons showed that there were fewer errors for *E-E* than for *E-I* (F(1, 22) = 11.06, p<0.01) and *I-I* (F(1, 22) = 5.66, p<0.05), which did not differ from each other. A correlation analysis for total RTs and errors within each condition showed that the only significant correlation was a positive one in the *E-E* length 3 condition (r = 0.77, p<0.01). This indicates that the results of RTs were not due to a speed-accuracy trade-off, which would imply a significant negative correlation between RTs and errors.

**Figure 2:**
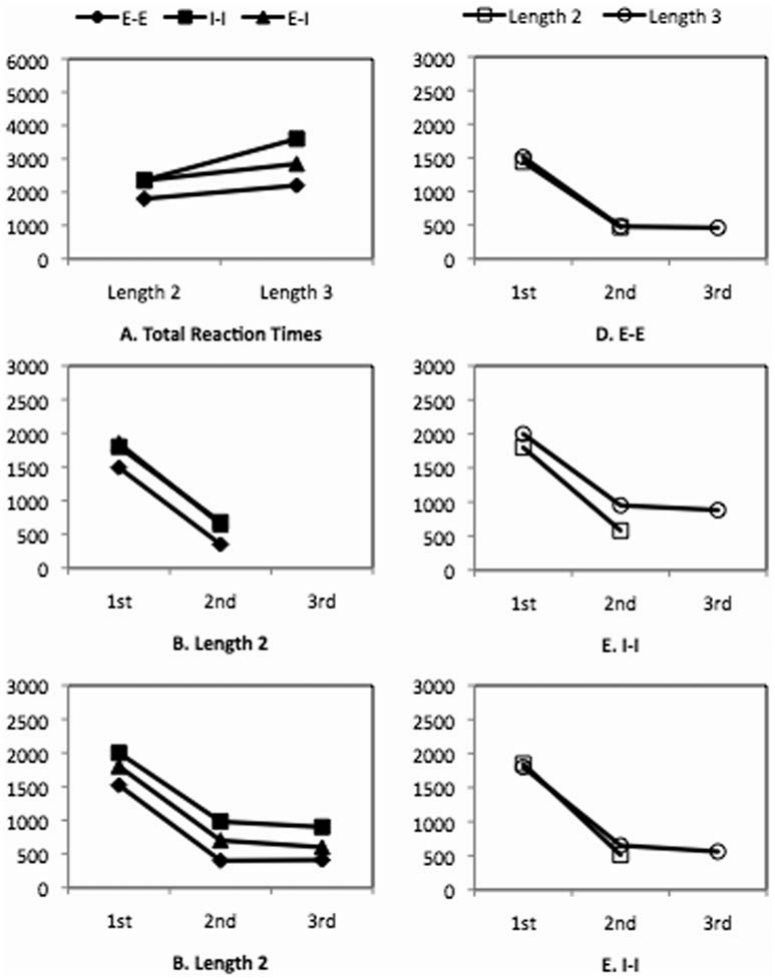
Reaction times (in ms) of Experiment 1, in which participants were asked to remember digits in Display 1 column by column, from top to bottom. 1st, 2nd, and 3rd indicate the order of individual comparisons.

The total RT for all individual comparisons in a trial is shown in Figure 2A for each condition. A two-way ANOVA for the three representations and two column lengths showed a significant interaction (F(2, 44) = 9.56, p<0.001), a significant length effect (F(1, 22) = 18.10, p<0.001), and a significant representation effect (F(2, 44) = 62.66, p<0.001). The interaction between lengths and representations for *E-E* and *I-I* was significant (F(1, 22) = 11.29, p<0.01), indicating that the increase of column length from 2 to 3 produced a larger RT increase for *I-I* than for *E-E*. Similarly, the interaction between lengths and representations for *E-I* and *I-I* was also significant (F(1, 22) = 13.29, p<0.001), indicating that the increase of column length from 2 to 3 produced a larger RT increase for *I-I* than for *E-I*. However, the interaction between lengths and representations for *E-I* and *E-E* was not significant, indicating that the increase of column length from 2 to 3 did not produce different RT increases for *E-I* and *E-E*.

Separate analyses were carried out for representations and lengths. For length 2, the total RT for *E-E* was significantly smaller than that for *I-I* (F(1, 11) = 13.56, p<0.01) and that for *E-I* (F(1, 11) = 39.36, p<0.001), which did not differ from each other. For length 3, the total RT for *E-E* was significantly smaller than that for *I-I* (F(1, 11) = 86.90, p<0.001) and that for *E-I* (F(1, 11) = 47.32, p<0.001), and the total RT for *E-I* was significantly smaller than that for *I-I* (F(1, 11) = 22.33, p<0.001).

In sum, the total RTs result in two findings. First, the difficulty orders were, from hardest to easiest, *I-I*≈*E-I*>*E-E* for length 2, and *I-I*>*E-I*>*E-E* for length 3. Second, with the increase of column length from 2 to 3, the RT increase for *I-I* was larger than that for *E-I* and that for *E-E*, which did not differ from each other.

The total RT for each trial was decomposed into individual RTs for the comparisons at different positions. As shown in Figure 2B and 2C, the RTs at the first position for both length 2 and length 3 were substantially larger than latter positions. This is expected because the RTs for the first comparison included initiation latencies, such as the time for selecting the two target columns (for all three representations). As a result, we only considered the individual RTs for the later positions. For length 2, the effect of representations was significant at the second position (F(2, 22) = 7.52, p<0.01). Separate comparisons showed that the RT for *E-E* was smaller than that for *I-I* (F(1, 11) = 9.65, p<0.01) and that for *E-I* (F(1, 11) = 7.49, p < 0.05), which did not differ from each other. For length 3, a two-way ANOVA for the three representations and the two positions (second and third) showed that the interaction was not significant, the RT at the second position was larger than that at the third position (F(1, 11) = 15.26, p < 0.01), and the effect of representations was significant (F(2, 22) = 39.65, p < 0.001). Simple comparisons of representations showed the following order of RTs: *I-I*>*E-I*>*E-E* (smallest F(1, 11) = 18.43 with largest p < 0.001).

Although it is not informative to compare the RTs at the first positions across different representations, it is informative to carry out a two-way ANOVA for the two column lengths and the first and second positions within a representation. This is because the initiation latencies for length 2 and length 3 should be the same for the same representation. Figures 2D, 2E, and 2F show the effects of column lengths on RTs for each representation. For both *E-E* and *E-I*, the interaction between lengths and positions was not significant, nor was the effect of lengths. For *I-I*, the interaction was not significant, but RTs for length 3 were marginally larger than those for length 2 (F(1, 22) = 3.75, p = 0.06).

In sum, the analyses of individual RTs result in the following findings. First, the difficulty order was, from hardest to easiest, *I-I*≈*E-I*>*E-E* for length 2, and *I-I*>*E-I*>*E-E* for length 3. This was consistent with the result for total RTs. Second, the individual RTs at the second and third positions were different across different representations, and the RTs decreased slightly from the second to the third position.

#### Summary

The finding that the individual RTs at the second and third positions were different across different representations indicates that participants adopted a step-by-step comparison strategy in which they made a single response after each pair of digits were compared, rather than an end-of-sequence comparison strategy in which they made all the responses at the end of sequence after all pairs of digits had been compared. However, the strategies for retrieving the pairs of digits for individual comparisons were different for different representations. Specifically, for both E-E and E-I, the retrieval of to-be-compared digits for each individual comparison was a pair-by-pair shifting process. This was expected for E-E because in this condition both target columns were available externally and directly accessible – participants simply had to shift their attention to the next pair of digits directly after each comparison. For E-I a pair-by-pair shifting was possible due to the column-by-column encoding strategy adopted for Display 1. Simply by conducting a digit-by-digit scanning in working memory, participants could retrieve a target digit from working memory and compare it with the corresponding digit on the screen, then shift to the next digit in working memory and compare it with next digit on the screen, until they finished all comparisons.

In contrast, digit-by-digit shifting was unlikely in *I-I* because when the digit in one column was retrieved, the corresponding digit in the other column were not immediately available: it had to be retrieved by sequentially scanning through other digits in working memory due to the column-by-column encoding. This was consistent with the finding that the RT for each individual comparison was marginally larger for length 3 than for length 2 in *I-I* but not in *E-I* and *E-E*. This length effect might be caused by the higher cost of memory scanning for the length 3 condition than for the length 2 condition. That is, in retrieving the two digits for each comparison participants had to search more digits in the length 3 condition than in the length 2 condition, resulting in larger RTs of individual comparisons for the former than for latter condition.

In summary, this experiment showed the following difficulty order: *I-I*≥*E-I*>*E-E*. This difficulty order means that given the same amount of information, when more information was available externally and less information was in working memory, the task became easier. However, the decrease of task difficulty with more information available externally was not because the capacity of working memory was limited. Rather, it was because the time for retrieving the digits for each comparison was decreased when information was available externally. This decrease of retrieval time was mainly due to the fact that the digits for each comparison in *I-I* could not be retrieved directly in a digit-by-digit shifting manner. Rather, they had to be retrieved by a sequential scanning through all the digits in working memory. This scanning, contributed to the difficulty order *I-I*≥*E-I*.

### Experiment 2

One major result of Experiment 1 suggests that the difficulty with internal representations in working memory has to do with the strict sequential memory scanning resulting from the column-to-column encoding. Experiment 2 further examines how the encoding of digits in working memory affects the performance and whether it changes the difficulty order for the three types of representations. Experiment 2 was identical to Experiment 1 except that the encoding of digits in working memory was different. Instead of memorizing the digits column by column from the top to the bottom, participants in Experiment 2 were instructed to memorize the digits on Display 1 (see Figure 2) *column by column in a reversed order*, that is, from the bottom to the top digit on the left column, then from the bottom to the top digit on the right column. Because *E-E* does not depend on how the digits are encoded in working memory, its results should be identical to those of the same condition in Experiment 1. The results of *I-I* of the current experiment should be similar to those of the same condition of Experiment 1 because the reversed order does not change the column-by-column encoding therefore still supports the direct retrieval of the two digits for each comparison. The *E-I* of the current experiment, however, should be different from the same condition in Experiment 1. In Experiment 1, a simple digit-by-digit shifting process would allow a straightforward match with external representations. In Experiment 2, in contrast, because the bottom-to-top reversed order of encoding does not directly support top-to-bottom digit-by-digit shifting, participants may have to resort to an extensive scanning process.

#### Method

22 undergraduate students in introductory psychology courses at The Ohio State University participated in the experiment for course credit. The study was approved by the institutional review board of The Ohio State University and written informed consent was obtained from each participant. The stimulus, design, and procedure were the same as in Experiment 1, except for the instructions for memorizing the digits on Display 1. In this experiment, participants were instructed to memorize the digits in Display 1 column by column in a reversed order, that is, memorize the left column first from the bottom digit to the top digit, then memorize the right column from the bottom digit to the top digit. To ensure that participants memorized the digits in the instructed order, the digits were presented one at a time, 500 ms per digit, starting from the bottom digit to the top digit of the left column, then the bottom digit to the top digit of the right column. The required response pattern was still from the top row to the bottom row.

#### Results

Similar procedures used in Experiment 1 were used here to pre-process the data.

The error rates for *E-E*, *E-I*, and *I-I* were 1.7%, 6.3%, and 5.1% for length 2, and 1.1%, 15.9%, and 20.5% for length 3. An ANOVA for representations and lengths showed a significant interaction (F(2, 40) = 6.31, p<0.01) and significant effects of lengths (F(1, 20) = 13.25, p<0.01) and representations (F(2, 40) = 14.57, p<0.001). For length 2, there were fewer errors for *E-E* than for *I-I* (F(1, 10) = 12.00, p<0.01) and *E-I* (F(1, 10) = 13.91, p<0.01), which did not differ from each other significantly. Similarly, for length 3, there were fewer errors for *E-E* than for *I-I* (F(1, 10) = 20.64, p<0.001) and *E-I* (F(1, 10) = 11.70, p<0.01), which did not differ from each other significantly. There were more errors for length 3 than length 2 for *I-I* (F(1, 20) = 14.46, p<0.01) and for *E-I* (F(1, 20) = 5.18, p<0.05), but there was no difference in errors between length 3 and length 2 for *E-E*. A correlation analysis for total RTs and errors within each condition showed that all correlations were positive and none of them was significant (strongest r = 0.46, p>0.10). This indicates that the results of RTs were not due to a speed-accuracy trade-off, which would imply a significant negative correlation between RTs and errors.

**Figure 3:**
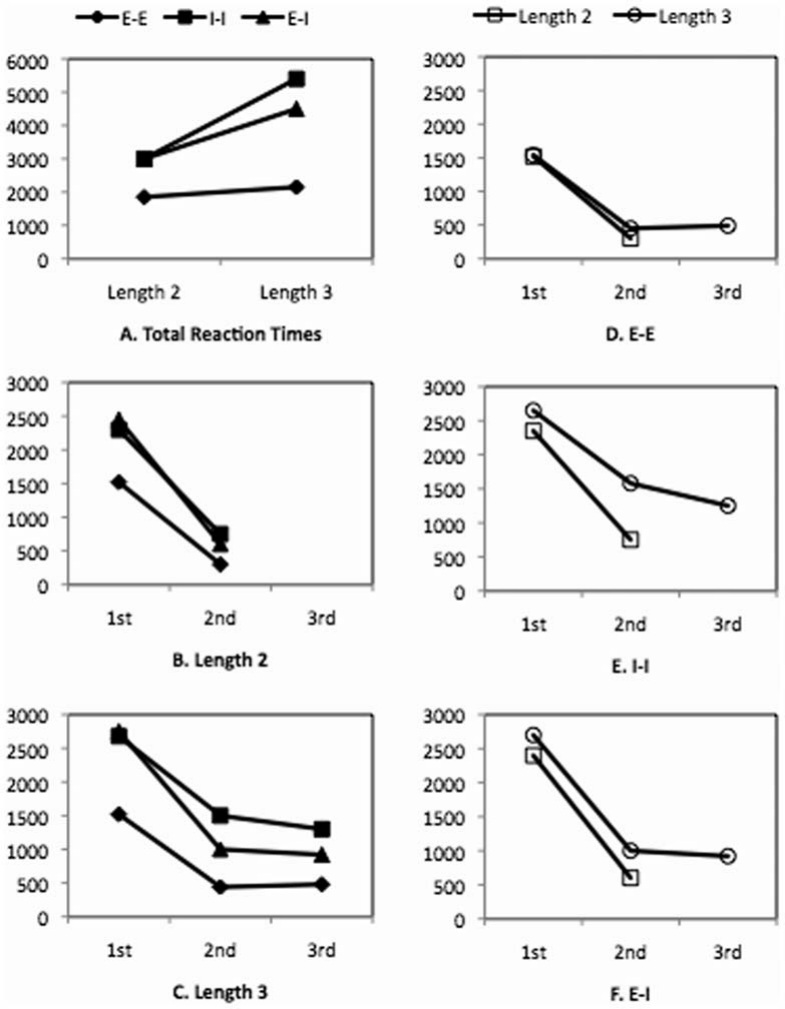
Reaction times (in ms) of Experiment 2, in which participants were asked to remember digits in Display 1 column by column, from bottom to top. 1st, 2nd, and 3rd indicate the order of individual comparisons.

In sum, the results of total RTs indicate two findings. First, the difficulty orders were, from hardest to easiest, *I-I*≈*E-I*>*E-E* for length 2, and *I-I*>*E-I*>*E-E* for length 3. Second, with the increase of column length from 2 to 3, the RT increase for *I-I* was larger than that for *E-I*, which in turn was larger than that for *E-E*.

The individual RTs are shown in Figures 3B and 3C. For length 2, the effect of representations was significant at the second position (F(2, 20) = 7.78, p<0.01). Separate analyses showed the RT at the second position for *E-E* was smaller than that for *I-I* (F(1, 10) = 10.10, p<0.01) and that for *E-I* (F(1, 10) = 25.62, p<0.001), which did not differ from each other significantly. For length 3, a two-way ANOVA for the three representations and the second and third positions showed a significant interaction (F(2, 20) = 7.07, p<0.01), a marginally significant effect of positions (F(1, 10) = 4.73, p<0.07), and a significant effect of representations (F(2, 20) = 36.95, p<0.001). Separate analyses for each position showed that for both the second and third positions, the differences between all representations were significant (smallest F(1, 10) = 15.01 with largest p<0.01). Separate analyses for each representation showed that the RT at the second position was larger than that at the third position for *I-I* (F(1, 10) = 11.03, p<0.01), and the RTs at the second and third positions did not differ from each other for *E-I* and *E-E.*

Similar to Experiment 1, an ANOVA for the two lengths and the first and second positions was carried out for each representation (see Figures 3D, 3E, and 3F). For *E-E*, the interaction between length and position was not significant, nor was the effect of lengths. For *I-I* and *E-I*, the interaction was not significant, but the effect of lengths was significant (F(1, 20) = 6.78, p<0.05; F(1, 20) = 7.27, p<0.01, respectively).

In sum, the analyses of individual RTs suggest the following results. First, the difficulty orders were, from hardest to easiest, *I-I*≈*E-I*>*E-E* for length 2, and *I-I*>*E-I*>*E-E* for length 3. This was consistent with the result for total RTs. Second, the individual RTs at the second and third positions were different across different representations, and the RTs decreased slightly from the second to the third position for *I-I* but not for *E-I* and *E-E*.

#### Summary

The main interest of Experiment 2 was to examine the effect of different encoding instructions on performance. We predicted that encoding Display 1 column-by-column in a reversed order would affect E-I but have no effect on E-E and I-I. This is indeed what we found. Results from both E-I and I-I in Experiment 2 were quite consistent with the corresponding conditions in Experiment 1 (see Figure 2D and 3D, Figure 2E and 3E). In both conditions, a pair-by-pair shifting process could be directly adopted.

The effect of our manipulation was mainly shown in *E-I*. Comparing Figure 2F and 3F, it is clear that the two experiments gave rise to different patterns – while the retrieval of digits for each individual comparison depended on column lengths in Experiment 2 it did not in Experiment 1. We argue that the length effect in the current experiment was because different encoding instructions led to different internal representations in working memory, which in turn led to different scanning processes in later problem solving. In Experiment 1, participants could use a digit-by-digit shifting process to retrieve the digit in working memory for each comparison because the response order was consistent with the encoding order, both from the top to the bottom. In the current experiment, however, the response order was from the top to the bottom but the encoding order was from the bottom to the top. Thus, to make the first comparison, participants needed to scan through all the digits in working memory to retrieve the top digit. Therefore, more digits in a column caused a larger RT for length 3 condition than for length 2 condition, resulting in the length effect.

In summary, this experiment showed the same difficulty order as in Experiment 1: *I-I*≥*E-I*>*E-E*. This difficulty order again indicates that given the same amount of information, when more information was distributed as external representations and less information was available in working memory, the task became easier. However, as demonstrated by the different result pattern in the *E-I* condition, it is clear that the efficiency of problem solving not only depends on the distribution of information among different sources, but also on the compatibility and coordination of information from these sources.

### Experiment 3

Experiments 1 and 2 both showed that the more information as external representations, the easier the task. In addition, Experiment 2 showed that problem solving performance was related to the compatibility of information in external representations and working memory. The purpose of Experiment 3 is to further test this hypothesis and show that the difficulty order might not hold if the coordination condition of information from different sources was changed. Experiment 3 was identical to Experiment 1 and 2 except that the encoding of digits on Display 1 (see Figure 2) was further manipulated. Instead of memorizing the digits column-by-column, participants in Experiment 3 were instructed to memorize the digits on Display 1 *row-by-row* from the top to the bottom, that is, memorize the top digit in the left column and the top digit in the right column, until the bottom digit in the left column and the bottom digit in the right column. With this row-by-row encoding, we predict the following results. First, because *E-E* does not depend on how the digits are encoded in working memory, its results should be still identical to those of the same condition in Experiment 1 and 2. Second, we expect the results from *I-I* and *E-I* would change. Specifically, in *I-I* of the current experiment, instead of using a sequential scanning strategy, the participants might use a more straightforward pair-by-pair shifting strategy for digit retrieval because the encoding order is the same as the response order. In *E-I* of the current experiment, participants might still use a pair-by-pair shifting strategy for digit retrieval. However, because only one of the two columns of digits in working memory contains the target digits, the non-target digits in the other column in working memory might prevent a straightforward pair-by-pair shifting. Therefore, as a result of the row-by-row encoding, we might not only get different retrieval strategies, but also get a different difficulty order, which was likely to be *E-I*>*I-I*>*E-E*. This difficulty order would suggest that more external representations do not always aid problem solving.

#### Method

24 undergraduate students in introductory psychology courses at The Ohio State University participated in the experiment for course credit. The study was approved by the institutional review board of The Ohio State University and written informed consent was obtained from each participant. The stimulus, design, and procedure were the same as in Experiment 1, except of the instructions for memorizing the digits on Display 1. In this experiment, participants were instructed to memorize the digits on Display 1 row by row, that is, memorize the top digit in the left column and the top digit in the right column, until the bottom digit in the left column and the bottom digit in the right column. The required response pattern was still from the top to the bottom pair (row).

#### Results

Similar procedures used in Experiment 1 were used here to pre-process the data.

The error rates for *E-E*, *E-I*, and *I-I* were 0.96%, 4.3%, and 2.4% for length 2, and 3.8%, 10.1%, and 5.3% for length 3. An ANOVA for representations and lengths showed a significant length effect (F(1, 24) = 10.80, p<0.01), a significant representation effect (F(2, 48) = 3.82, p<0.05), and a non-significant interaction. Simple comparisons of representations showed that there were significantly more errors for *E-I* than for *E-E* (F(1, 24) = 8.96, p<0.01), but there were no significant differences in errors between *E-I* and *I-I* and between *I-I* and *E-E*. A correlation analysis for total RTs and errors within each condition showed that the only significant correlation was a positive one in the *I-I* length 3 condition (r = 0.65, p = 0.02). This indicates that the results of RTs were not due to a speed-accuracy trade-off, which would imply a significant negative correction between RTs and errors.

**Figure 4:**
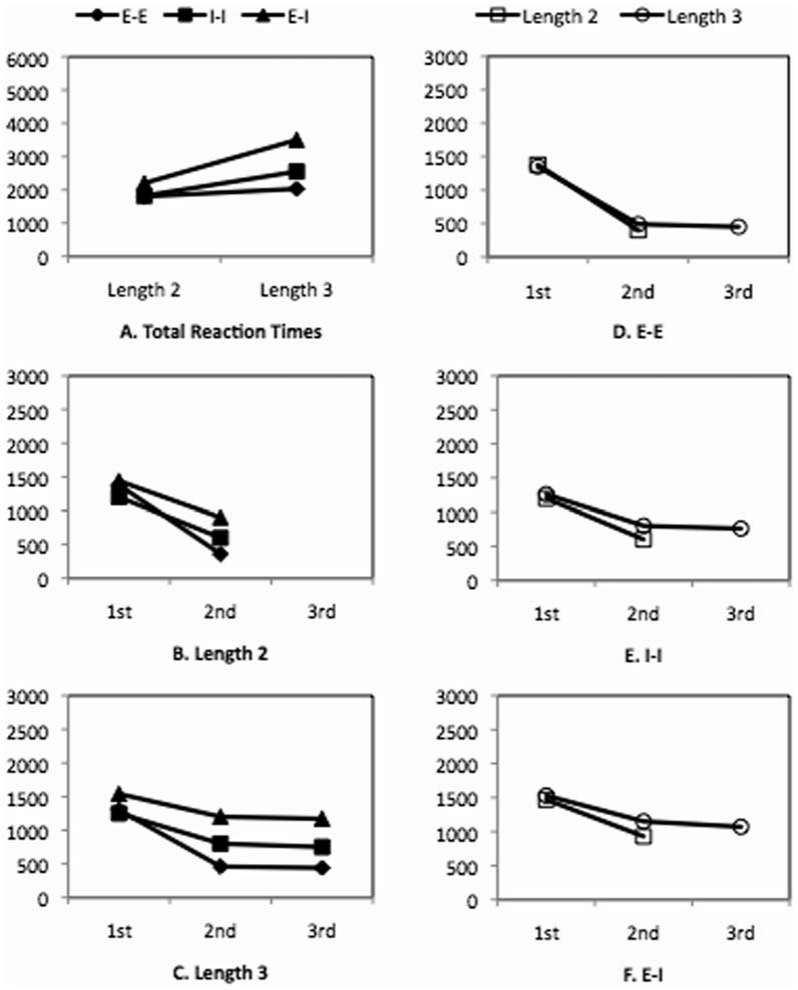
Reaction times (in ms) of Experiment 3, in which participants were asked to remember digits in Display 1 row by row. 1st, 2nd, and 3rd indicate the order of individual comparisons.

The total RTs are shown in Figure 4A. An ANOVA for lengths and representations showed a significant interaction (F(2, 48) = 14.23, p<0.001), a significant length effect (F(1, 24) = 20.19, p<0.001), and a significant representation effect (F(2, 48) = 75.52, p<0.001). For every pair of representations, the interaction between lengths and representations was significant (smallest F(1, 20) = 5.96 with largest p<0.05). This indicates that the increase of column length from 2 to 3 produced a larger RT increase for *E-I* than for *E-E* and for *I-I*, and a larger RT increase for *I-I* than for *E-E*. Separate analyses were conducted for representations and lengths. For length 2, the total RT for *E-I* was larger than that for *E-E* (F(1, 12) = 60.87, p<0.001) and that for *I-I* (F(1, 12) = 43.50, p<0.001), which did not differ from each other significantly. For length 3, the total RT for *E-I* was significantly larger that that for *I-I* (F(1, 12) = 54.17, p<0.001) and that for *E-E* (F(1, 12) = 68.82, p<0.001), and the total RT for *I-I* was significantly larger than that for *E-E* (F(1, 12) = 11.56, p<0.01).

In sum, these results lead to two findings. First, the difficulty orders were, from hardest to easiest, *E-I*>*I-I*≈*E-E* for length 2, and *E-I*>*I-I*>*E-E* for length 3. These difficulty orders were different from those in Experiments 1 and 2, which were *I-I*≥*E-I*>*E-E*. Second, with the increase of column length from 2 to 3, the RT increase for *E-I* was larger than that for *I-I*, which in turn was larger than that for *E-E*. This was also different from that in Experiments 1 and 2, in which the RT increase for *I-I* was larger than that for *E-I*, which in turn was larger than that for *E-E*.

The individual RTs are shown in Figures 4B and 4C. For length 2, the effect of representations was significant at the second position (F(2, 24) = 29.65, p<0.001). Separate analyses showed the following order of RTs at the second position: *E-I*>*I-I*>*E-E* (smallest F(1, 12) = 19.99 with largest p<0.001). For length 3, a two-way ANOVA for the three representations and the second and third positions showed that a significant position effect (F(1, 12) = 7.65, p<0.05), a significant representations effect (F(2, 24) = 52.88, p<0.001), but a non-significant interaction. Separate comparisons for representations showed the following order of RTs for the second and third positions: *E-I*>*I-I*>*E-E* (smallest F(1, 12) = 27.96 with largest p<0.001).

A two-way ANOVA for the two column lengths and the first and second positions was carried out for each representation (Figures 4D, 4E, and 4F). For all three representations, none of the interactions between lengths and positions were significant, nor were the effects of lengths.

In sum, the analyses of individual RTs suggest the following results. First, the difficulty order was, from hardest to easiest, *E-I*>*I-I*>*E-E* for both length 2 and length 3. Second, the individual RTs at the second and third positions were different across different representations, and the RTs decreased slightly from the second to the third position.

#### Summary

In Experiment 3, we changed the encoding of digits on Display 1 to be row-by-row. The effect of this manipulation was clear. As predicted, while the results for E-E were consistent with those in the previous experiments the results for I-I and E-I were different. In I-I of the current experiment, the retrieval of digits did not depend on column lengths, that is, in retrieving the two digits for each comparison participants did not search more digits in length 3 than in length 2 condition (see Figure 4E). This non-significant length effect suggests that the retrieval of digits for each comparison was a pair-by-pair shifting process, supported directly by the row-by-row encoding in working memory. In E-I, the retrieval of digits for each individual comparison also did not depend on column lengths (see Figure 4F). However, the existence of intermediary non-target digits in the target column, resulting from the row-by-row encoding, clearly interfered with the retrieval of those target digits. In particularly, because the non-target digit in working memory occupied the same position as the target digit in external representations, it could also interfere with the processing of the target digit in external representations. As a result of the interference, it took longer to compare a pair of digits in E-I than a pair of digits in I-I.

In summary, this experiment showed a difficulty order of *E-I*>*I-I*>*E-E*, which was different from that in Experiments 1 and 2. In particular, the task in which one column of digits was in external representations and the other was in working memory was more difficult than the task in which both columns of digits were in working memory, due to the different representational structures of two sources and the resulting interference from such incompatibility. This difficulty order was inconsistent with the general claim that the more information in external representations, the easier the task, suggesting that external representations do not always enhance task performance.

## Discussion

The three experiments carried out in the present study explicitly test the memory aid hypothesis of external representations: external representations serve as memory aids and improve task performance by means of expanding the limited capacity of working memory. The results, using a sequential number comparison task, show that external representations could hinder as well as enhance task performance. Specifically, a task with all information in external representations is easier than a task with part or all information in working memory, but a task with information distributed across working memory and external representations can be easier or harder, depending on how the information from different sources is compatible and coordinated. In particular, when external and internal representations are incompatible, either due to the specific encoding of internal representations or the specific presentation format of external representations, the task performance may be negatively affected. Hence, the memory aid hypothesis of external representations should not be taken for granted.

The results from the current study highlight the complex relations between external representations and working memory. First, external representations are separate from internal representations in working memory in that external representations need not to be re-represented internally in working memory in order to be used. This is supported by the different retrieval strategies for external representations and working memory. Our experiments show that *E-E* was always easier than *I-I* regardless of how the digits in working memory were encoded. The explicit separation of external representations and working memory is consistent with the view of situated cognition [18]–[24], which argues that it is not necessary to construct an internal model of the external environment to perform cognitive tasks: people can directly access the situational information in the external environment and act upon it in an adaptive manner. It is also consistent with the view of distributed representations [6], [7], [9]–[11], which argues that the representation of a cognitive task involving external representations is neither solely internal nor solely external, but distributed as a system of distributed representations with internal and external representations as two indispensable parts.

Second, when the task requires information from both external representations and working memory, it is the interaction of information from the two sources that determines the task difficulty. This is consistent with a line of research in cognitive science that distinguishes processes and representations. We show that when information from the two sources matches well for the task requirement, the task becomes easier in that one does not have to re-organize or re-process the information in working memory, as demonstrated in Experiment 1. On the other hand, when information from the two sources does not match well for the task requirement, one has to re-process the information in working memory, either through a time-consuming sequential search or re-representation, resulting in decreased task performance. In our Experiment 2, the column-by-column but reversed-order encoding made *E-I* length-dependent. In our Experiment 3, the row-by-row encoding actually damaged the performance in *E-I* and made it harder than *I-I* even though more information was available externally in the former condition.

Although our results support the general claim that task performance is determined by the coordination of information from external representations and working memory, it is important to note that in our experiments we only manipulated the different encoding strategies used for representations in working memory but kept external representations constant. A large body of research in this area has demonstrated extensively, by manipulating different types of external representations, that external representations play an important role in affecting task performance [6], [7]. Taken together, these results support the conclusion that while both external and internal representations are important, it is the coordination among information from both sources that is more critical in distributed problem solving.

## Acknowledgments

We would like to thank Richard Carlson and Krishna Tateneni for their suggestions and comments and Dwen Hall for his assistance in carrying out the experiments.
